# The Association Between Attachment Style and Symptomatic, Social, and Personal Recovery: A Comparison Between Male and Female Patients Remitted From Their First‐Episode Psychosis

**DOI:** 10.1111/eip.70152

**Published:** 2026-03-13

**Authors:** Justine de With, Kit van der Eng, Iris E. Sommer, Marieke van der Pluijm, Frederike Schirmbeck, Lieuwe de Haan, HAMLETT and OPHELIA Consortium Investigators, Iris Sommer, Lieuwe de Haan, Wim Veling, Jim van Os, Filip Smit, Marieke Begemann, Sanne Schuite‐Koops, Machteld Marcelis, Martijn Kikkert, Nico van Beveren, Nynke Boonstra, Bram‐Sieben Rosema, P Roberto Bakker, Sinan Gülöksüz, Joran Lokkerbol, Bodyl Brand, Shiral Gangadin, Chris Geraets, Erna Van’t Hag, Priscilla Oomen, Alban Voppel, Therese van Amelsvoort, Maarten Bak, Agaath Been, Marinte van den Bosch, Truus van den Brink, Gunnar Faber, Koen Grootens, Martin de Jonge, Henderikus Knegtering, Jörg Kurkamp, Amrita Mahabir, Gerdina Hendrika Maria Pijnenborg, Tonnie Staring, Wiek Vaes, Natalie Veen, Selene Veerman, Sybren Wiersma

**Affiliations:** ^1^ Department of Psychiatry Amsterdam UMC (Location AMC) Amsterdam the Netherlands; ^2^ Department of Biomedical Sciences of Cells and Systems, and Department of Psychiatry University of Groningen, University Medical Center Groningen Groningen the Netherlands; ^3^ Arkin Institute for Mental Health Amsterdam the Netherlands; ^4^ Department of Public Mental Health Central Institute of Mental Health, Medical Faculty Mannheim, Heidelberg University Mannheim Germany

**Keywords:** attachment style, clinical recovery, first‐episode psychosis, personal recovery, psychosis, recovery outcome, schizophrenia, sex, social recovery

## Abstract

**Background:**

Sex differences in recovery outcomes for psychosis have been observed. In patients with psychosis, rates of insecure attachment are significantly higher in patients of both sexes compared to the general population and have been linked to several aspects of recovery. However, the possible differential effect of attachment style on recovery between men and women with psychosis is currently unknown.

**Methods:**

This study was performed in a subsample of 299 patients remitted from their first‐episode psychosis (FEP) within the Handling Antipsychotic Medication Long‐term Evaluation of Targeted Treatment (HAMLETT) study. First, *t*‐tests were used to explore sex differences in baseline attachment style. Second, stepwise regression analyses were used to examine the association between baseline attachment style and symptomatic, social and personal recovery at three and 48 months follow‐up, and the possible moderation effect of sex on these associations. Third, stepwise regression analyses were repeated with longitudinal change in symptomatic, social and personal recovery between three and 48 months follow‐up as outcome measure.

**Results:**

Male and female patients did not differ in baseline attachment style. Baseline attachment style was associated with recovery outcome at 3‐months follow‐up, whilst sex did not moderate this relationship. Baseline attachment style did not predict recovery outcome at 48‐months follow‐up, nor change in recovery outcome.

**Conclusions:**

Our findings suggest that attachment style is an important predictor of short‐term recovery outcome in patients with FEP, whilst sex differences do not appear to significantly impact this relationship.

## 1 Introduction

Sex in first‐episode psychosis (FEP) is increasingly recognised as a predictor of the age of onset, premorbid (social) functioning, course of symptoms and recovery outcomes (Cotton et al. 2009; Ochoa et al. 2012; Fernando et al. 2020; Sommer et al. 2020; Thorup et al. 2014). Men have a higher risk to develop psychosis and an earlier age of onset compared to women (Li et al. 2022). Men experience more severe negative symptoms and cognitive decline, whilst women experience more affective symptoms (Riecher‐Rössler et al. 2018; Brand et al. 2022). Moreover, women demonstrate higher premorbid functioning and superior outcomes compared to men during the early stages of illness (Riecher‐Rössler et al. 2018; Brand et al. 2022). However, recent studies have shown that this advantageous start for women within the first years diminishes over time, resulting in comparable rates of recovery between genders during later stages of illness (Brand et al. 2022; Sommer et al. 2020; Dubreucq et al. 2021).

Attachment style is a concept that may help understand how sex‐specific differences manifest in the course of illness in FEP as attachment style reflects the impact of early relational experiences on factors related to recovery such as interpersonal functioning, emotion regulation and resilience (van Bussel et al. 2023). The attachment theory describes the importance of an early childhood secure emotional bond between the infant and the primary attachment figures (Bowlby 1969; Ainsworth and Bell 1970). The infant is biologically determined to seek protection and comfort from parents in moments of stress, threat, pain, or distress. If the primary attachment figures respond predictably, reliably, and responsively enough, the reassuring and comforting experiences with the parents are internalised into a secure or autonomously stable attachment style (Bowlby 1969; Ainsworth and Bell 1970). However, if the parents are insufficiently able to regulate their child's distress, insecure attachment patterns emerge. This might result in emotion regulation deficits later in life and during adulthood. Two insecure attachment style dimensions are distinguished: insecure‐anxious and insecure‐avoidant attachment (Brennan et al. 1998). An anxious attachment style is characterised by emotional dysregulation and reduced sense of autonomy leading to elevated dependence on others. Avoidant attachment is best illustrated by an avoidance of close relationships and over‐regulation of emotions. A large previous meta‐analysis in the general population showed that sex differences in attachment style become apparent around late childhood and are more prominently observed during adulthood (del Giudice 2011). Men are more prone to insecure‐avoidant attachment, whereas women display more insecure‐anxious attachment styles (del Giudice 2011; Ciocca et al. 2020; Weber et al. 2022; Paquette et al. 2001; Schmitt 2003).

In patients with psychosis, significantly higher rates of insecure attachment are found compared to non‐clinical samples (Carr et al. 2018). This is already detected in early stages of the disease, both in ultra‐high risk (Quijada et al. 2015) and FEP samples (Couture et al. 2007). Several systematic reviews reported associations between attachment style and psychotic symptomatology (Carr et al. 2018; Berry et al. 2007; Korver‐Nieberg et al. 2014; Gumley, Taylor, et al. 2014; van Bussel et al. 2021), and specifically paranoia (Lavin et al. 2020; Sood et al. 2022; Murphy et al. 2020). In addition, attachment insecurity has been linked to poorer recovery outcomes (Berry et al. 2007; Korver‐Nieberg et al. 2014; van Bussel et al. 2021; Pearse et al. 2020). More specifically, a recent meta‐analysis including 17 studies of patients with psychosis (including 4 FEP samples) found that higher scores on anxious and avoidant attachment were cross‐sectionally related to worse symptomatic, social or personal recovery (van Bussel et al. 2021). Individual cross‐sectional FEP studies show contradicting results as some studies reported associations between insecure attachment and recovery (Couture et al. 2007; Korver‐Nieberg et al. 2013; Gumley, Schwannauer, et al. 2014; Fett et al. 2016), whereas others did not (MaBeth et al. 2011; Berry et al. 2018). Although the number of prospective studies is very limited, there is some preliminary evidence suggesting that insecure attachment is associated with symptomatic or functional recovery in patients with schizophrenia (van Bussel et al. 2021; Berry et al. 2008; Pearse et al. 2020), FEP (Gumley, Schwannauer, et al. 2014), or those at risk‐mental state (Quijada et al. 2015). There are no studies investigating the associations between attachment style and personal recovery over time.

Despite the growing body of literature suggesting links between attachment style and recovery outcome, little is known about sex differences in attachment style in patients with psychosis and findings are inconsistent (Couture et al. 2007; Mulligan and Lavender 2010; Wickham et al. 2015; MaBeth et al. 2011). One study reported higher rates of anxious attachment and lower rates of avoidant attachment in men with psychosis compared to female patients (Couture et al. 2007), whilst other studies found the opposite (Mulligan and Lavender 2010) or did not find sex differences at all (Wickham et al. 2015; Arbuckle et al. 2012; Aydin et al. 2016; MaBeth et al. 2011). Moreover, we are not aware of a previous cross‐sectional or longitudinal study investigating sex differences in attachment style and associations with recovery outcomes in patients with psychosis.

Hence, the first aim of the present study was to investigate sex differences in baseline attachment style in a large sample of patients remitted from FEP. We hypothesised to find higher levels of avoidant attachment in men and higher levels of anxious attachment in women, in line with findings in the general population (del Giudice 2011; Ciocca et al. 2020; Weber et al. 2022; Paquette et al. 2001). Our second aim was to explore the relationship between baseline attachment style and symptomatic, social and personal recovery at 3 and 48 months follow‐up and investigate whether sex would moderate this relationship. We hypothesised that higher scores on anxious and avoidant attachment would predict worse recovery at 3 and 48 months. As we expected that avoidant attachment styles are more prevalent among men, we hypothesised a stronger association between baseline avoidant attachment and recovery outcomes in men compared to women. Similarly, as we expected that anxious attachment styles are more prevalent among women, we hypothesised a stronger association between baseline anxious attachment and recovery outcomes in women compared to men. Our last aim was to explore the association between baseline attachment style and change in symptomatic, social and personal recovery over time and investigate whether sex would moderate this relationship. Again, we hypothesised to find sex differences affecting the strength of the associations in men and women.

## 2 Method

### 2.1 Study Design and Participants

Data from the Handling Antipsychotic Medication Long‐term Evaluation of Targeted Treatment (HAMLETT) study were used. HAMLETT is a Dutch multi‐site pragmatic single‐blind randomised controlled trial designed to study the long‐term effects of continuation versus dose‐reduction/discontinuation of antipsychotic medication after remission of FEP. Inclusion criteria were an age range from 16 to 55 years, mastery of the Dutch language and use of antipsychotic medication. Patients had to meet the criteria for FEP and reach symptomatic remission three to 6 months before the baseline visit. Symptomatic remission was evaluated using the CASH (Andreasen et al. 1992) and using the MINI Screener (developed at the department of Psychiatry, UMC Groningen). In total, 432 participants were included at baseline. Included patients were randomised to continuation of antipsychotic medication until at least 1 year after remission or gradual dose reduction according to a tapering schedule. The study was approved by the Medical Ethics Committee (METC) of the University Medical Center Groningen (METC‐number 2017‐343). Written informed consent was obtained from participants before they were enrolled in the study. The full procedure of the study has been described elsewhere (Begemann et al. 2020). There was no singular measurement point at which data for attachment style, symptomatic recovery, social recovery, and personal recovery were gathered. Therefore, in the current study, attachment style data was derived from the second visit (baseline) and combined with data for symptomatic recovery, social recovery, and personal recovery from the third visit (3 months after baseline) and eighth visit (48 months after baseline, see Table S1). We included patients with data on these predictor and outcome measures, leading to a subsample of 299 patients.

### 2.2 Measurements

#### 2.2.1 Sex

Sex was determined based on self‐reported information; patients were asked about their sex assigned at birth.

#### 2.2.2 Attachment Style

Attachment style was measured with the Dutch version of the Psychosis Attachment Measure (PAM) (Korver 2014). The Dutch PAM is a 15‐item self‐report questionnaire with seven items reflecting avoidant attachment and eight reflecting anxious attachment. Items are rated on a 4‐point Likert rating scale, rating from 0 (not at all) to 3 (very much) (Korver 2014). Mean item scores were calculated for avoidant and anxious attachment, with higher scores reflecting higher levels of insecure attachment (Berry et al. 2006; Korver 2014). The Dutch PAM is observed to be an adequate measurement with high construct validity and reliability in a clinical and non‐clinical sample. Chronbach's alpha of the two PAM domains ranged from 0.70 to 0.83 (Korver 2014).

#### 2.2.3 Symptomatic Recovery

*S*everity of psychotic symptoms was assessed with the Positive and Negative Syndrome Scale (PANSS) (Kay et al. 1988). The PANSS is a 30‐item clinician‐rated interview assessing positive symptoms, negative symptoms and general psychopathology over the past week. Items are rated on a 7‐point Likert scale, with higher scores reflecting a higher severity of symptoms. A total sum score was calculated.

#### 2.2.4 Social Recovery

Social recovery was measured with the Dutch version of the World Health Organization's Disability Assessment Schedule 2.0 (WHODAS 2.0) (Ustün et al. 2010). The WHODAS 2.0 is a 36‐item questionnaire covering six domains of functioning in everyday life. As we aimed to investigate social recovery (rather than general functioning), we only included the WHODAS 2.0 domains that reflect social functioning; ‘getting along’ and ‘participation’. The ‘getting along’ domain assesses difficulties in interactions with other people that might be encountered due to illness. The ‘participation’ domain assesses the limitations on activity and restrictions on participation in society someone experienced due to illness. Items are rated on a 5‐point Likert scale and scored using item‐response‐theory based scoring, which takes multiple levels of difficulty for each item into account (Ustün et al. 2010). Higher domain sum scores indicate a lower level of social functioning. The WHODAS 2.0 has high internal consistenty (Chronbach's alpha coefficient of 0.96.) and good test–test reliability (intraclass correlation coefficient of 0.98) (Ustün et al. 2010).

#### 2.2.5 Personal Recovery

Personal recovery was measured with the Dutch version of the Recovery Assessment Scale (RAS) (van der Krieke et al. 2019). This self‐report questionnaire assesses the degree of which an individual experiences personal recovery through 24 items that have resulted from the factor analysis of the original scale consisting of 30 items (van der Krieke et al. 2019). Items are rated on a 5‐point Likert rating scale ranging from 1 (strongly disagree) to 5 (strongly agree), with higher scores reflecting higher levels of personal recovery. A mean total score was calculated. The Dutch RAS has demonstrated high internal consistency with a Chronbach's alpha coefficient of 0.90 (van der Krieke et al. 2019). Since the response option ‘not applicable’ was mistakenly added to three items that assumed the presence of symptoms, these three items were recoded. Therefore, response option 6 (not applicable) was converted to 5 (strongly agree) as these answer option seems to be most closely aligned (see Table S2). To assess whether recoding of the three items affect the outcome of our analyses, additional post hoc analyses were performed to compare the results with the ‘not applicable’ answer option recoded as 5 (strongly agree) versus when the ‘not applicable’ answer option was labelled as missing item.

#### 2.2.6 Covariates

To correct for possible confounding effects, the covariates age, illness duration, level of education, and childhood trauma were a priori selected based on previously reported associations with attachment style (Carr et al. 2018; Gumley, Taylor, et al. 2014; Pearse et al. 2020). Age at onset was based on the Comprehensive Assessment of Symptoms and History (CASH) (Andreasen et al. 1992). Illness duration (time from diagnosis to remission) and level of education were evaluated using a self‐reported questionnaire specifically developed for the HAMLETT study. Level of education was categorised into low, medium, and higher education (Luijkx and de Heus 2008). Childhood trauma was assessed using the Childhood Trauma Questionnaire Short Form (CTQ‐SF), a 25‐item self‐report questionnaire (Bernstein et al. 2003). A total trauma sum score was calculated based on all individual items combined, with higher scores representing more severe trauma exposure.

### 2.3 Statistical Analyses

All statistical analyses were performed using Statistical Package for the Social Sciences (IBM SPSS Statistics) version 28.0. The assumption of normality was checked visually for the most important measures (PAM, RAS and WHODAS 2.0) using P–P and Q–Q plots of standardised residuals.

In the first analysis, differences between male and female patients regarding attachment style at baseline were compared by independent *t*‐tests.

In the second analysis, stepwise multiple regression analyses were performed to explore the possible moderating effect of sex on the association between attachment style and symptomatic, functional and personal recovery, whilst correcting for the covariates age, illness duration, level of education and childhood trauma. In a first step, all covariates were entered en bloc. In a second step, sex and attachment style were entered. Finally, in a third step, sex*attachment style interaction effects were added to investigate possible moderating effects of sex. All analyses were run separately for anxious and avoidant attachment and the recovery outcome measures.

In the third analysis, the differential impact of baseline attachment style on change in symptomatic, functional and personal recovery over time between male and female patients was examined. Therefore, we calculated change scores for the three recovery outcome measures over a 45 months period (3 months to 48 months). Again, stepwise multiple regression analysis was performed in the aforementioned three steps. Multicollinearity was tested by calculating the correlation matrix, the Variance Inflation Factor (VIF), and the Tolerance statistic for the variables included in the models in step 1 and 2. No serious multicollinearity existed among attachment style and the a priori selected covariates. Since four different outcome variables were tested (symptomatic recovery, two social recovery domains and personal recovery), we used a Bonferroni correction to minimise the risk of type I errors. Thus, the two‐tailed significance threshold was set at 0.0125 (0.05/4).

## 3 Results

### 3.1 Sample Characteristics

Complete data on attachment style (baseline) was available for 202 men and 97 women. For available data on the recovery outcome measures see Table S3. Differences in baseline demographic characteristics between men and women are shown in Table 1. Male patients were significantly younger and had a longer illness duration at baseline. The distribution of ethnicity and educational level was significantly different between included male and female patients. For detailed information on recovery outcome scores at 3 and 48 months follow‐up see Table S4.

**TABLE 1:** Baseline characteristics of included male and female patients.^a^

	Male (*n* = 202)	Female (*n* = 97)	Test statistic^b^	*p*
Age in years (SD)	27.29 (8.39)	30.30 (9.77)	*t*(276) = −2.547	0.011*
Ethnicity *N*(%)			X (1) = 4.845	0.028*
The Netherlands	186 (91.18%)	80 (82.47%)		
Other	18 (8.82%)	17 (17.53%)		
Education *N*(%)			X (2) = 10.879	0.004*
Low	24 (11.88%)	4 (4.12%)		
Medium	95 (47.03%)	35 (36.08%)		
High	83 (41.09%)	58 (59.79%)		
DSM V diagnosis *N*(%)			X (7) = 9.371	0.227
Schizophrenia	12 (6.09%)	4 (4.35%)		
Schizophreniform disorder	14 (7.11%)	4 (4.35%)		
Schizoaffective disorder	3 (1.52%)	4 (4.35%)		
Delusional disorder	9 (4.57%)	3 (3.26%)		
Brief psychotic disorder	27 (13.71%)	22 (23.91%)		
Substance induced psychotic disorder	8 (4.06%)	1 (1.09%)		
Unspecified psychotic Disorder	84 (42.64%)	38 (41.30%)		
Other specified psychotic Disorder	40 (20.30%)	16 (17.39%)		
Illness duration in weeks (SD)	33.85 (94.10)	16.50 (13.91)	*t*(207.249) = 2.491	0.014*
Childhood trauma (SD)	37.47 (9.00)	40.34 (13.79)	*t*(119.520) = −1.749	0.083

Abbreviation: SD, standard deviation.

^a^ Childhood trauma was collected at 3‐month follow up.

^b^ Independent *t*‐test for continuous data and Pearson *X* ^2^ for categorical data.

* *p*‐value < 0.05.

### 3.2 Sex Differences in Attachment Style at Baseline

We did not find differences between male and female patients regarding attachment styles at baseline (see Table 2).

**TABLE 2:** Sex differences in baseline attachment style.

Attachment style (PAM)	Male	Female	Test statistic	*p*
Anxious attachment	1.70 (0.52)	1.75 (0.55)	*t*(295) = −0.737	0.462
Avoidant attachment	2.22 (0.53)	2.17 (0.54)	*t*(299) = 0.877	0.381

*Note:* Data are in mean (standard deviation).

Abbreviation: PAM, Psychosis Attachment Measure.

### 3.3 Sex Differences in the Association Between Attachment Style and Recovery Outcome Measures at 3 and 48 Months

Due to missing data on prospective outcome measures, analyses were conducted in a smaller subsample (see Table S3). The regression models were carried out in three steps: step 1 (model with covariates), step 2 (added main effects of sex and attachment style), step 3 (added interaction effect). Table 3 presents the final model step 3. Step 1 and 2 are presented as Supporting Information (see Table S5). Anxious attachment was significantly associated with all recovery outcome domains when correcting for covariates at 3‐months follow‐up, also after correcting for multiple testing. Avoidant attachment was significantly associated with clinical recovery, the social recovery domain getting along, and personal recovery at 3‐months follow‐up when accounting for covariates, although the association with clinical recovery lost significance after correcting for multiple testing. Added interaction effects were not significant, hence no moderating effect for sex was found for any of the outcome measures. There were no significant associations between attachment style and recovery outcome at 48‐months follow‐up.

**TABLE 3:** Sex differences in the association between baseline attachment style and symptomatic, functional and personal recovery at 3 and 48 months, whilst correcting for covariates (step 3).

Symptomatic recovery (PANSS)						
	3‐month follow‐up			48‐month follow‐up		
	*β* (SE)	*p*	*R* ^2^	*β* (SE)	*p*	*R* ^2^
PAM anxiety						
Constant	32.505 (4.558)	< 0.001	0.108	51.557 (13.511)	< 0.001	0.127
Age	0.116 (0.075)	0.124		−0.052 (0.152)	0.735	
Illness duration	0.000 (0.009)	0.977		0.108 (0.092)	0.242	
Education	−2.371 (1.044)	0.024*		−2.551 (2.605)	0.332	
Childhood trauma	0.100 (0.065)	0.124		0.134 (0.154)	0.390	
Sex	2.104 (4.879)	0.667		−13.646 (12.557)	0.282	
Anxiety	5.109 (1.743)	0.004**		−4.753 (4.816)	0.328	
Sex* anxiety	−1.657 (2.678)	0.537		5.844 (6.944)	0.404	
PAM avoidance						
Constant	32.446 (4.964)	< 0.001	0.079	49.405 (12.607)	< 0.001	0.125
Age	0.083 (0.076)	0.272		−0.064 (0.151)	0.673	
Illness duration	0.007 (0.008)	0.353		0.018 (0.018)	0.298	
Education	−1.965 (1.026)	0.057		−2.179 (2.608)	0.407	
Childhood trauma	0.129 (0.069)	0.062		0.115 (0.149)	0.444	
Sex	7.793 (5.982)	0.194		−20.701 (13.299)	0.125	
Avoidance	3.321 (1.681)	0.050*		−1.993 (3.840)	0.606	
Sex* avoidance	−3.711 (2.668)	0.166		7.905 (5.964)	0.190	

Social functioning: getting along (WHODAS 2.0)						
	3‐month follow‐up			48‐month follow‐up		
	*β* (SE)	*p*	*R* ^2^	*β* (SE)	*p*	*R* ^2^
PAM anxiety						
Constant	−12.435 (7.510)	0.099	0.138	−7.307 (22.580)	0.747	0.181
Age	0.008 (0.124)	0.950		−0.374 (0.258)	0.153	
Illness duration	−0.009 (0.015)	0.547		0.199 (0.156)	0.207	
Education	0.754 (1.720)	0.662		−1.810 (4.304)	0.676	
Childhood trauma	0.264 (0.106)	0.014*		0.622 (0.263)	0.021*	
Sex	−6.757 (8.038)	0.402		5.384 (21.546)	0.804	
Anxiety	7.993 (2.871)	0.006**		6.895 (8.190)	0.403	
Sex* anxiety	2.758 (4.411)	0.532		−7.006 (11.928)	0.559	
PAM avoidance						
Constant	−16.119 (8.528)	0.060	0.113	9.815 (21.131)	0.644	0.151
Age	−0.063 (0.130)	0.627		−0.419 (0.258)	0.109	
Illness duration	0.022 (0.013)	0.107		−0.012 (0.030)	0.696	
Education	1.719 (1.764)	0.331		−1.800 (4.331)	0.679	
Childhood trauma	0.299 (0.118)	0.012**		0.605 (0.256)	0.021*	
Sex	2.529 (10.270)	0.806		−11.378 (22.890)	0.621	
Avoidance	6.737 (2.887)	0.021*		0.096 (6.620)	0.988	
Sex* avoidance	−1.590 (4.575)	0.729		2.131 (10.295)	0.837	

Social functioning: participation (WHODAS 2.0)						
	3‐month follow‐up			48‐month follow‐up		
	*β* (SE)	*p*	*R* ^2^	*β* (SE)	*p*	*R* ^2^
PAM anxiety						
Constant	−7.978 (7.785)	0.307	0.116	−18.004 (20.876)	0.392	0.150
Age	0.212 (0.129)	0.102		−0.144 (0.239)	0.549	
Illness duration	0.005 (0.016)	0.739		0.054 (0.144)	0.709	
Education	0.222 (1.782)	0.901		−0.722 (3.979)	0.857	
Childhood trauma	0.186 (0.110)	0.092		0.638 (0.243)	0.011**	
Sex	−2.906 (8.332)	0.728		27.830 (19.920)	0.168	
Anxiety	7.747 (2.976)	0.010**		7.618 (7.572)	0.318	
Sex* anxiety	2.025 (4.572)	0.658		−13.885 (11.027)	0.213	
PAM avoidance						
Constant	−5.341 (8.376)	0.524	0.056	6.967 (19.285)	0.719	0.164
Age	0.153 (0.128)	0.233		−0.170 (0.235)	0.473	
Illness duration	0.009 (0.013)	0.505		0.031 (0.028)	0.262	
Education	1.139 (1.732)	0.512		0.114 (3.952)	0.977	
Childhood trauma	0.261 (0.115)	0.025*		0.598 (0.234)	0.013*	
Sex	2.989 (2.835)	0.293		−5.391 (6.042)	0.376	
Avoidance	13.821 (10.086)	0.172		−9.060 (20.891)	0.666	
Sex* avoidance	−5.615 (4.493)	0.213		5.943 (9.396)	0.529	

Personal recovery (RAS)						
	3‐month follow‐up			48‐month follow‐up		
	*β* (SE)	*p*	*R* ^2^	*β* (SE)	*p*	*R* ^2^
PAM anxiety						
Constant	4.811 (0.222)	< 0.001	0.222	3.832 (0.723)	< 0.001	0.110
Age	−0.010 (0.004)	0.006		0.000 (0.009)	0.988	
Illness duration	0.000 (0.000)	0.979		0.005 (0.005)	0.326	
Education	0.130 (0.051)	0.011**		−0.054 (0.143)	0.706	
Childhood trauma	−0.007 (0.003)	0.026*		−0.010 (0.009)	0.265	
Sex	−0.097 (0.238)	0.683		1.245 (0.719)	0.091	
Anxiety	−0.384 (0.085)	< 0.001**		0.265 (0.295)	0.374	
Sex* anxiety	0.087 (0.130)	0.504		−0.550 (0.396)	0.172	
PAM avoidance						
Constant	5.047 (0.234)	< 0.001	0.246	3.935 (0.770)	< 0.001	0.102
Age	−0.007 (0.004)	0.037*		0.001 (0.010)	0.924	
Illness duration	0.000 (0.000)	0.675		0.004 (0.005)	0.426	
Education	0.079 (0.048)	0.106		−0.047 (0.146)	0.750	
Childhood trauma	−0.005 (0.003)	0.168		−0.009 (0.009)	0.280	
Sex	−0.155 (0.282)	0.582		1.216 (0.785)	0.129	
Avoidance	−0.423 (0.080)	< 0.001**		0.141 (0.235)	0.553	
Sex* avoidance	0.074 (0.126)	0.555		−0.424 (0.345)	0.226	

Abbreviations: PANSS, Positive and Negative Syndrome Scale; *R* ^2^, coefficient of determination; RAS, Recovery Assessment Scale; WHODAS, WHO Disability Assessment Schedule.

* *p*‐value < 0.05.

** *p* < 0.0125. Considering correction for multiple testing.

### 3.4 Sex Differences in the Prospective Association Between Attachment Style and Change in Recovery Outcome Measures Over Time

The differential impact of baseline attachment style on change in symptomatic, functional and personal recovery over 45 months (3–48 months) between male and female patients was evaluated using linear regression models. For detailed information on the number of included patients see Table S6. Again, regression models were carried out in three steps (the covariates age, illness duration, education and childhood trauma entered at step 1; sex and attachment style entered at step 2; sex*attachment style interaction effects entered at step 3). Attachment style did not significantly predict change in recovery over time, nor was there a moderation effect of sex (see Table S7).

## 4 Discussion

In the current study, we examined sex differences in baseline attachment style in patients with FEP of the multicentre HAMLETT study and in the relationship between attachment style and different recovery measures. More specifically, we investigated sex differences in the association between baseline attachment style and symptomatic, social and personal recovery at 3 and 48 months, and moreover in the association between baseline attachment style and change in symptomatic, social and personal recovery over these months. No significant differences were observed in baseline attachment style between male and female patients. Across patients with psychosis, attachment style predicted short‐term recovery outcome, whilst sex did not appear to significantly impact this relationship. Attachment style was not found to predict long‐term recovery outcome.

Contradicting our hypothesis, no significant sex differences were observed in baseline anxious and avoidant attachment scores between male and female FEP patients. This finding is consistent with some previous studies in patients with FEP (MaBeth et al. 2011) or schizophrenia spectrum disorders (Wickham et al. 2015; Arbuckle et al. 2012; Aydin et al. 2016). However, other studies in FEP (Couture et al. 2007; Mulligan and Lavender 2010) and in the general population (del Giudice 2011; Ciocca et al. 2020; Weber et al. 2022; Paquette et al. 2001) did find sex differences. One explanation for these discrepancies might be that the majority of studies reporting sex differences (Ciocca et al. 2020; Weber et al. 2022; Paquette et al. 2001; Couture et al. 2007; Mulligan and Lavender 2010; del Giudice 2011; Schmitt 2003) included larger sample sizes than the studies that did not (Wickham et al. 2015; Arbuckle et al. 2012; Aydin et al. 2016; MaBeth et al. 2011), hence had more power to detect relatively small effects. Yet, our study is also large, but still finds no sex difference. Another explanation might be a higher variance in attachment style in the general population compared to our patient sample.

To the best of our knowledge, this is the first study investigating the relationship between attachment style and both short‐term and long‐term recovery outcomes in patients with FEP, considering a broad spectrum of recovery. The positive associations we found between attachment style and short‐term recovery in individuals with FEP are in accordance with cross‐sectional findings in patients with schizophrenia spectrum disorders in previous reviews (Berry et al. 2007; Korver‐Nieberg et al. 2014; van Bussel et al. 2021; Pearse et al. 2020) and in a previous meta‐analysis (van Bussel et al. 2021). Our study confirms previous results and contributes new insight regarding short‐term prospective associations across different stages of psychosis by including patients remitted from their FEP. As far as we know, only one previous study investigated the association between attachment style and psychotic symptoms prospectively in patients with FEP. Gumley, Schwannauer, et al. (2014) showed that attachment style predicted recovery of negative symptoms 6 months and 12 months later. Our finding of a short‐term effect of attachment style on recovery outcome, with no observed long‐term effect suggests that attachment style may exert an initial influence on recovery, which is subsequently overshadowed by other factors that emerge over the longer term. However, the absence of a significant association between attachment style and recovery outcome 48 months later in our study, can be attributed to our relatively small sample size, which might have limited the power to detect relatively small associations at 48 months. This might also apply to the influence of attachment style on change of recovery over time. Given this limitation, our findings should be interpreted cautiously. Future prospective studies focusing on longitudinal associations are needed to confirm our findings. Nevertheless, our results highlight the importance of addressing attachment style in the early stages of psychosis. Hence, research should evaluate whether early targeted interventions on improving attachment style enhance recovery outcome.

Contrary to our expectations, our results revealed that sex did not moderate the found associations between attachment style and recovery outcome. Since this is the first study investigating sex differences in this association, caution in drawing conclusions is warranted. The predictors included in our analyses left considerable variance unexplained. This might suggest that other important social determinants (related to attachment style) may play a prominent role in determining recovery outcome. These social determinants may be similar in men and women but could also vary by sex. One determinant we hypothesised to play an important role is childhood trauma. Indeed, in the present study experiencing trauma in childhood seemed to have a confounding effect on recovery outcome. However, after adding attachment style to our models, the unique contribution of childhood trauma reduced. Since collinearity between these constructs was limited, this might indicate that attachment style serves as a stronger or more direct predictor of recovery outcome than childhood trauma. However, shared influence cannot be ruled out. Another explanation for the absence of a moderation effect of sex was suggested by del Giudice (2009), hypothesising that in particular the variability in the male and female distribution of psychological variables is different with men showing greater variability. Since in the present study the standard deviations of attachment styles were equal in men and women, this hypothesis does not hold for attachment style in our sample. Moreover, it is possible that sex differences manifest more profoundly in fluctuations over time, which is interesting to assess further.

The present study expands upon prior research in several ways. We are the first to asses sex differences in attachment style in relation to recovery at two prospective time points and over time. Moreover, we investigated a broad scope of recovery by selecting clinical, social and personal recovery. Aside from limitations previously noted, it is important to consider some other limitations when interpreting our findings. First, self‐report questionnaires have been used to assess attachment style, social recovery and personal recovery, which may introduce the possibility of self‐report biases (Olbert et al. 2016). Second, we cannot rule out that when we measure attachment style, a perceived sex specific rol pattern is incorporated. We doubt whether this is a limitation, as this has still societal relevance. Third, we classified patients based on their sex at birth, neglecting the possible influence of gender identity. However, as in our sample only one of the 299 included patients reported that gender identity differed from biological sex, it was not possible to incorporate both sex and gender. Fourth, restricting our sample to only FEP patient in symptomatic remission has limited the generalizability of our findings. Therefore, future studies should aim to replicate these results in a more diverse FEP population including patients who are not (yet) in symptomatic remission. Fifth, we relied on attachment style as stable construct over time as this is widely assumed (Waters et al. 2000; Chris Fraley 2002). Nonetheless, it is conceivable that attachment can change. Notably, Berry et al. (2008) showed that change in anxious attachment (but not avoidant attachment), was positively correlated with change in symptom severity in patients with psychosis over a 6 month period. An important difference from our study, however, is that the patients in the study of Berry et al. (2008) received psychotherapy which increases the likelihood of a change in attachment style. Generalizability of our findings to all patients with schizophrenia spectrum disorder may be limited, since we included patients who remitted from their first psychotic episode and who consented to participate in a challenging study. Future longitudinal studies are warranted to test whether attachment style evolves across illness duration and therapy. It is widely assumed that women with psychosis have a later age of onset and higher educational level compared to men (Ochoa et al. 2012; Thorup et al. 2014), therefore we assume that our sample reflects differences in the population. Lastly, patients included in the present study were randomly assigned to either continue their antipsychotic medication or to taper it gradually. This was not taken into account as a variable in the present analyses due to the single‐blind, ongoing nature of the HAMLETT. Nevertheless future research may incorporate this factor, especially in light of preliminary evidence suggesting a link between attachment style and treatment adherence (McGonagle et al. 2021).

In conclusion, we observed no differences in attachment style between men and women in patients remitted from their FEP. We showed that attachment style is an important predictor of short‐term recovery outcome in patients with FEP, whilst sex differences do not appear to significantly impact this relationship. Our findings emphasise the importance of addressing attachment style when treating both male and female patients with recent onset psychosis. Further longitudinal studies are warranted to test whether attachment style evolves across illness duration and therapy and how this relates to clinical, social and personal recovery.

## Funding

The HAMLETT study is funded by ZonMW in the Netherlands (grant number. 80‐84 800‐98‐41015). The funders have no role in the study design, collection, management, analysis and interpretation of data, writing the report, or the decision to submit the report for publication.

## Ethics Statement

The authors assert that all procedures contributing to this work comply with the ethical standards of the relevant national and institutional committees on human experimentation and with the Helsinki Declaration of 1975, as revised in 2008.

## Conflicts of Interest

The authors declare no conflicts of interest.

## Supporting information


**Table S1:** Overview of available data per assessment time of the included patients.
**Table S2:** Detailed information on recoded items of the Recovery Assessment Scale (RAS).
**Table S3:** Overview of patients included in the prospective analyses regarding the association between baseline attachment style and recovery outcomes at 3 and 48 months follow up.
**Table S4:** Clinical recovery outcome scores at 3 and 48 months follow up of included male and female patients.
**Table S5:** Sex differences in the association between baseline attachment style and symptomatic, functional and personal recovery at 3 and 48 months.
**Table S6:** Overview of patients included in the prospective analyses regarding the association between baseline attachment style and change in recovery outcomes over time (between 3 and 48 months follow‐up).
**Table S7:** Sex differences in the prospective association between baseline attachment style and change in recovery outcome measures over time whilst correcting for covariates.

## Data Availability

Research data are not shared.
