# Supplementary material for: The Association Between Attachment Style and Symptomatic, Social, and Personal Recovery: A Comparison Between Male and Female Patients Remitted From Their First‐Episode Psychosis
Source: Early Interv Psychiatry. 2026 Mar 13;20(3):e70152. doi: 10.1111/eip.70152 (PMC12985686; doi:10.1111/eip.70152)
Supplement: Supplementary file 1 — Table S1: Overview of available data per assessment time of the included patients. Table S2: Detailed information on recoded items of the Recovery Assessment Scale (RAS). Table S3: Overview of patients included in the prospective analyses regarding the association between baseline attachment style and recovery outcomes at 3 and 48 months follow up. Table S4: Clinical recovery outcome scores at 3 and 48 months follow up of included male and female patients. Table S5: Sex differences in the association between baseline attachment style and symptomatic, functional and personal recovery at 3 and 48 months. Table S6: Overview of patients included in the prospective analyses regarding the association between baseline attachment style and change in recovery outcomes over time (between 3 and 48 months follow‐up). Table S7: Sex differences in the prospective association between baseline attachment style and change in recovery outcome measures over time whilst correcting for covariates. [file EIP-20-0-s001.docx]

**Supplementary appendix**

This appendix is supplement to:

*The association between attachment style and symptomatic, social, and personal recovery: a comparison between male and female patients remitted from their first-episode psychosis*

**Index**:

**Supplement 1.** Overview of available data per assessment time of the included patients.

**Supplement 2.** Detailed information on recoded items of the Recovery Assessment Scale (RAS).

**Supplement 3.** Overview of patients included in the prospective analyses regarding the association between baseline attachment style and recovery outcomes at 3 and 48 months follow up.

**Supplement 4.** Clinical recovery outcome scores at 3 and 48 months follow up of included male and female patients.

**Supplement 5.** Sex differences in the prospective association between baseline attachment style and symptomatic, functional, and personal recovery at 3 and 48 months, while correcting for covariates.

**Supplement 6.** Overview of patients included in the prospective analyses regarding the association between baseline attachment style and change in recovery outcomes over time (between 3 and 48 months follow up).

**Supplement 7.** Sex differences in the prospective association between baseline attachment style and change in recovery outcome measures over time.

**Supplement 8.** Sex differences in the prospective association between baseline attachment style and change in recovery outcome measures over time, while correcting for covariates.

**Supplementary Table S1:** Overview of available data per assessment time of the included patients

| **Visit** | **V1** | **V2** | **V3** | **V4** | **V8** |
| --- | --- | --- | --- | --- | --- |
| Months | -3 tot 0 | 0 | 3 | 6 | 48 |
| Gender | X |  |  |  |  |
| PAM anxiety |  | X |  |  |  |
| PAM avoidance |  | X |  |  |  |
| WHODAS 2.0 getting along |  |  | X |  | X |
| WHODAS 2.0 participation |  |  | X |  | X |
| RAS |  |  | X |  | X |
| PANSS |  |  | X |  | X |
| Age | X |  |  |  |  |
| Illness duration | X |  |  |  |  |
| Medication | X |  |  |  |  |
| Education | X |  |  |  |  |
| Ethnicity | X |  |  |  |  |
| Childhood trauma |  |  | X |  |  |
| Abbreviations: PAM=Psychosis Attachment Measure; WHODAS 2.0=WHO Disability Assessment Schedule; RAS=Recovery Assessment Scale; PANSS=Positive and Negative Syndrome Scale. | | | | | |

**Supplementary Table S2:** Detailed information on recoded items of the Recovery Assessment Scale (RAS).

| 15. Coping with mental illness is no longer the main focus of my life  (1) Completely disagree  (2) Disagree  (3) Mixed  (4) Agree  (5) Completely agree  (6) Not applicable |
| --- |
| 16. My symptoms interfere less and less with my life  (1) Completely disagree  (2) Disagree  (3) Mixed  (4) Agree  (5) Completely agree  (6) Not applicable |
| 17. My symptoms seem to be a problem for shorter periods of time each time they occur (1) Completely disagree  (2) Disagree  (3) Mixed  (4) Agree  (5) Completely agree  (6) Not applicable |

**Supplementary Table S3:** Overview of patients included in the prospective analyses regarding the association between baseline attachment style and recovery outcomes at 3 and 48 months follow up.

|  | **PAM anxiety** | | **PAM avoidance** | |
| --- | --- | --- | --- | --- |
|  | *Male* | *Female* | *Male* | *Female* |
| Symptomatic recovery (PANSS) at 3-months | 194 | 93 | 196 | 95 |
| Symptomatic recovery (PANSS) at 48-months | 51 | 29 | 53 | 30 |
|  |  |  |  |  |
| Social recovery: getting along (WHODAS 2.0) at 3-months | 200 | 95 | 202 | 97 |
| Social recovery: getting along (WHODAS 2.0) at 48-months | 53 | 31 | 55 | 32 |
|  |  |  |  |  |
| Social recovery: participation (WHODAS 2.0) at 3-months | 200 | 95 | 202 | 97 |
| Social recovery: participation (WHODAS 2.0) at 48-months | 53 | 31 | 55 | 32 |
|  |  |  |  |  |
| Personal recovery (RAS) at 3-months | 171 | 86 | 174 | 88 |
| Personal recovery (RAS) at 48-months | 34 | 25 | 34 | 26 |
| Data are in numbers.  Abbreviations: PAM = Psychosis Attachment Measure; PANSS = Positive and Negative Syndrome Scale; WHODAS = WHO Disability Assessment Schedule; RAS = Recovery Assessment Scale. | | | | |

**Supplementary Table S4.** Clinical recovery outcome scores at 3 and 48 months follow up of included male and female patients.

| **3-months follow up** | **Male** | **Female** |
| --- | --- | --- |
| Symptomatic recovery (PANSS) | 43.46 (11.12) | 41.72 (8.71) |
| Social recovery (WHODAS) |  |  |
| Getting along | 14.42 (19.82) | 12.54 (17.06) |
| Participation | 19.30 (17.96) | 19.46 (17.67) |
| Personal recovery (RAS) | 3.90 (0.55) | 3.91 (0.56) |
|  | | |
| **48-months follow up** | **Male** | **Female** |
| Symptomatic recovery (PANSS) | 43.25 (11.65) | 38.43 (11.60) |
| Social recovery (WHODAS) |  |  |
| Getting along | 16.06 (23.23) | 10.94 (19.79) |
| Participation | 15.08 (19.49) | 16.80 (19.36) |
| Personal recovery (RAS) | 3.89 (0.70) | 4.07 (0.36) |
| Data are in mean (standard deviation).  Abbreviations: PANSS = Positive and Negative Syndrome Scale; WHODAS = WHO Disability Assessment Schedule ; RAS = Recovery Assessment Scale. | | |

**Supplementary Table S5.** Sex differences in the association between baseline attachment style and symptomatic, functional, and personal recovery at 3 and 48 months.

| **Symptomatic recovery (PANSS)** | | | | | | |
| --- | --- | --- | --- | --- | --- | --- |
|  | **3-month follow up** | | | **48-month follow up** | | |
| **PAM anxiety** | β (SE) | *p-value* | R^2^ | β (SE) | *p-value* | R^2^ |
| *Step 1* |  |  |  |  |  |  |
| Constant | 37.634 (4.101) | <0.001 | 0.061 | 45.093 (10.047) | <0.001 | 0.091 |
| Age | 0.111 (0.076) | 0.146 |  | -0.060 (0.150) | 0.692 |  |
| Illness duration | 0.002 (0.009) | 0.813 |  | 0.138 (0.088) | 0.124 |  |
| Education | -1.908 (1.042) | 0.068 |  | -3.372 (2.491) | 0.181 |  |
| Childhood trauma | 0.162 (0.062) | 0.009** |  | 0.096 (0.141) | 0.496 |  |
| *Step 2* |  |  |  |  |  |  |
| Constant | 33.566 (4.217) | <0.001 | 0.106 | 46.093 (11.819) | <0.001 | 0.116 |
| Age | 0.118 (0.075) | 0.118 |  | -0.044 (0.152) | 0.771 |  |
| Illness duration | 0.000 (0.009) | 0.983 |  | 0.117 (0.091) | 0.201 |  |
| Education | -2.342 (1.042) | 0.026* |  | -2.824 (2.578) | 0.278 |  |
| Childhood trauma | 0.098 (0.065) | 0.131 |  | 0.161 (0.151) | 0.290 |  |
| Sex | -0.785 (1.420) | 0.581 |  | -3.427 (3.189) | 0.287 |  |
| Anxiety | 4.462 (1.393) | 0.002** |  | -1.956 (3.476) | 0.576 |  |
| *Step 3* |  |  |  |  |  |  |
| Constant | 32.505 (4.558) | <0.001 | 0.108 | 51.557 (13.511) | <0.001 | 0.127 |
| Age | 0.116 (0.075) | 0.124 |  | -0.052 (0.152) | 0.735 |  |
| Illness duration | 0.000 (0.009) | 0.977 |  | 0.108 (0.092) | 0.242 |  |
| Education | -2.371 (1.044) | 0.024* |  | -2.551 (2.605) | 0.332 |  |
| Childhood trauma | 0.100 (0.065) | 0.124 |  | 0.134 (0.154) | 0.390 |  |
| Sex | 2.104 (4.879) | 0.667 |  | -13.646 (12.557) | 0.282 |  |
| Anxiety | 5.109 (1.743) | 0.004** |  | -4.753 (4.816) | 0.328 |  |
| Sex*anxiety | -1.657 (2.678) | 0.537 |  | 5.844 (6.944) | 0.404 |  |
| **PAM avoidance** |  |  |  |  |  |  |
| *Step 1* |  |  |  |  |  |  |
| Constant | 38.321 (4.047) | <0.001 | 0.060 | 48.098 (9.805) | <0.001 | 0.077 |
| Age | 0.096 (0.075) | 0.203 |  | -0.077 (0.150) | 0.610 |  |
| Illness duration | 0.010 (0.008) | 0.219 |  | 0.018 (0.017) | 0.299 |  |
| Education | -1.899 (1.014) | 0.062 |  | -3.539 (2.486) | 0.160 |  |
| Childhood trauma | 0.149 (0.061) | 0.016* |  | 0.093 (0.141) | 0.511 |  |
| *Step 2* |  |  |  |  |  |  |
| Constant | 35.355 (4.512) | <0.001 | 0.070 | 43.132 (11.759) | <0.001 | 0.099 |
| Age | 0.089 (0.076) | 0.239 |  | -0.060 (0.152) | 0.695 |  |
| Illness duration | 0.008 (0.008) | 0.299 |  | 0.014 (0.017) | 0.430 |  |
| Education | -1.811 (1.023) | 0.078 |  | -2.699 (2.595) | 0.303 |  |
| Childhood trauma | 0.113 (0.068) | 0.099 |  | 0.129 (0.150) | 0.392 |  |
| Sex | -0.286 (1.435) | 0.842 |  | -3.577 (3.172) | 0.264 |  |
| Avoidance | 2.025 (1.402) | 0.150 |  | 1.213 (3.001) | 0.688 |  |
| *Step 3* |  |  |  |  |  |  |
| Constant | 32.446 (4.964) | <0.001 | 0.079 | 49.405 (12.607) | <0.001 | 0.125 |
| Age | 0.083 (0.076) | 0.272 |  | -0.064 (0.151) | 0.673 |  |
| Illness duration | 0.007 (0.008) | 0.353 |  | 0.018 (0.018) | 0.298 |  |
| Education | -1.965 (1.026) | 0.057 |  | -2.179 (2.608) | 0.407 |  |
| Childhood trauma | 0.129 (0.069) | 0.062 |  | 0.115 (0.149) | 0.444 |  |
| Sex | 7.793 (5.982) | 0.194 |  | -20.701 (13.299) | 0.125 |  |
| Avoidance | 3.321 (1.681) | 0.050* |  | -1.993 (3.840) | 0.606 |  |
| Sex*avoidance | -3.711 (2.668) | 0.166 |  | 7.905 (5.964) | 0.190 |  |
|  |  | | | | |  |
| **Social functioning: getting along (WHODAS 2.0)** | | | | | | |
|  | **3-month follow up** | | | **48-month follow up** | | |
| **PAM anxiety** | β (SE) | *p-value* | R^2^ | β (SE) | *p-value* | R^2^ |
| *Step 1* |  |  |  |  |  |  |
| Constant | -5.820 (6.844) | 0.396 | 0.069 | 8.895 (16.765) | 0.598 | 0.150 |
| Age | -0.011 (0.127) | 0.932 |  | -0.427 (0.254) | 0.098 |  |
| Illness duration | -0.004 (0.016) | 0.788 |  | 0.186 (0.149) | 0.218 |  |
| Education | 1.562 (1.737) | 0.370 |  | -2.873 (4.096) | 0.486 |  |
| Childhood trauma | 0.394 (0.102) | <0.001** |  | 0.544 (0.240) | 0.027* |  |
| *Step 2* |  |  |  |  |  |  |
| Constant | -14.200 (6.949) | 0.042 | 0.137 | -0.884 (19.649) | 0.964 | 0.176 |
| Age | 0.005 (0.124) | 0.968 |  | -0.384 (0.256) | 0.139 |  |
| Illness duration | -0.009 (0.015) | 0.541 |  | 0.187 (0.154) | 0.229 |  |
| Education | 0.707 (1.715) | 0.681 |  | -1.435 (4.233) | 0.736 |  |
| Childhood trauma | 0.267 (0.106) | 0.012** |  | 0.590 (0.255) | 0.024* |  |
| Sex | -1.948 (2.335) | 0.405 |  | -6.878 (5.307) | 0.200 |  |
| Anxiety | 9.070 (2.294) | <0.001** |  | 3.591 (5.921) | 0.546 |  |
| *Step 3* |  |  |  |  |  |  |
| Constant | -12.435 (7.510) | 0.099 | 0.138 | -7.307 (22.580) | 0.747 | 0.181 |
| Age | 0.008 (0.124) | 0.950 |  | -0.374 (0.258) | 0.153 |  |
| Illness duration | -0.009 (0.015) | 0.547 |  | 0.199 (0.156) | 0.207 |  |
| Education | 0.754 (1.720) | 0.662 |  | -1.810 (4.304) | 0.676 |  |
| Childhood trauma | 0.264 (0.106) | 0.014* |  | 0.622 (0.263) | 0.021* |  |
| Sex | -6.757 (8.038) | 0.402 |  | 5.384 (21.546) | 0.804 |  |
| Anxiety | 7.993 (2.871) | 0.006** |  | 6.895 (8.190) | 0.403 |  |
| Sex*anxiety | 2.758 (4.411) | 0.532 |  | -7.006 (11.928) | 0.559 |  |
| **PAM avoidance** |  |  |  |  |  |  |
| *Step 1* |  |  |  |  |  |  |
| Constant | -5.804 (-0.043) | 0.409 | 0.083 | 14.499 (16.179) | 0.373 | 0.127 |
| Age | -0.043 (0.130) | 0.741 |  | -0.445 (0.254) | 0.085 |  |
| Illness duration | 0.027 (0.013) | 0.050* |  | -0.007 (0.029) | 0.806 |  |
| Education | 1.502 (1.754) | 0.393 |  | -3.455 (4.054) | 0.397 |  |
| Childhood trauma | 0.404 (0.106) | <0.001** |  | 0.5333 (0.240) | 0.030* |  |
| *Step 2* |  |  |  |  |  |  |
| Constant | -14.877 (7.727) | 0.056 | 0.113 | 8.163 (19.417) | 0.676 | 0.150 |
| Age | -0.061 (0.130) | 0.639 |  | -0.418 (0.256) | 0.107 |  |
| Illness duration | 0.022 (0.013) | 0.100 |  | -0.013 (0.030) | 0.656 |  |
| Education | 1.783 (1.751) | 0.310 |  | -1.941 (4.244) | 0.649 |  |
| Childhood trauma | 0.292 (0.116) | 0.012** |  | 0.609 (0.253) | 0.019* |  |
| Sex | -0.936 (2.455) | 0.703 |  | -6.768 (5.263) | 0.203 |  |
| Avoidance | 6.183 (2.402) | 0.011** |  | 0.956 (5.115) | 0.852 |  |
| *Step 3* |  |  |  |  |  |  |
| Constant | -16.119 (8.528) | 0.060 | 0.113 | 9.815 (21.131) | 0.644 | 0.151 |
| Age | -0.063 (0.130) | 0.627 |  | -0.419 (0.258) | 0.109 |  |
| Illness duration | 0.022 (0.013) | 0.107 |  | -0.012 (0.030) | 0.696 |  |
| Education | 1.719 (1.764) | 0.331 |  | -1.800 (4.331) | 0.679 |  |
| Childhood trauma | 0.299 (0.118) | 0.012** |  | 0.605 (0.256) | 0.021* |  |
| Sex | 2.529 (10.270) | 0.806 |  | -11.378 (22.890) | 0.621 |  |
| Avoidance | 6.737 (2.887) | 0.021* |  | 0.096 (6.620) | 0.988 |  |
| Sex*avoidance | -1.590 (4.575) | 0.729 |  | 2.131 (10.295) | 0.837 |  |
|  |  | | | | |  |
| **Social functioning: participation (WHODAS 2.0)** | | | | | | |
|  | **3-month follow up** | | | **48-month follow up** | | |
| **PAM anxiety** | β (SE) | *p-value* | R^2^ | β (SE) | *p-value* | R^2^ |
| *Step 1* |  |  |  |  |  |  |
| Constant | -2.152 (7.039) | 0.760 | 0.060 | -5.608 (15.488) | 0.718 | 0.120 |
| Age | 0.212 (0.131) | 0.106 |  | -0.153 (0.235) | 0.517 |  |
| Illness duration | 0.009 (0.016) | 0.590 |  | 0.013 (0.138) | 0.924 |  |
| Education | 1.228 (1.786) | 0.493 |  | 0.683 (3.784) | 0.857 |  |
| Childhood trauma | 0.322 (0.105) | 0.003** |  | 0.628 (0.222) | 0.006** |  |
| *Step 2* |  |  |  |  |  |  |
| Constant | -9.274 (7.200) | 0.199 | 0.115 | -5.275 (18.352) | 0.775 | 0.128 |
| Age | 0.210 (0.128) | 0.104 |  | -0.165 (0.240) | 0.492 |  |
| Illness duration | 0.005 (0.016) | 0.742 |  | 0.030 (0.144) | 0.834 |  |
| Education | 0.188 (1.777) | 0.916 |  | 0.020 (3.954) | 0.996 |  |
| Childhood trauma | 0.189 (0.110) | 0.087 |  | 0.573 (0.239) | 0.019* |  |
| Sex | 0.624 (2.420) | 0.797 |  | 3.530 (4.957) | 0.479 |  |
| Anxiety | 8.537 (2.376) | <0.001** |  | 1.071 (5.530) | 0.847 |  |
| *Step 3* |  |  |  |  |  |  |
| Constant | -7.978 (7.785) | 0.307 | 0.116 | -18.004 (20.876) | 0.392 | 0.150 |
| Age | 0.212 (0.129) | 0.102 |  | -0.144 (0.239) | 0.549 |  |
| Illness duration | 0.005 (0.016) | 0.739 |  | 0.054 (0.144) | 0.709 |  |
| Education | 0.222 (1.782) | 0.901 |  | -0.722 (3.979) | 0.857 |  |
| Childhood trauma | 0.186 (0.110) | 0.092 |  | 0.638 (0.243) | 0.011** |  |
| Sex | -2.906 (8.332) | 0.728 |  | 27.830 (19.920) | 0.168 |  |
| Anxiety | 7.747 (2.976) | 0.010** |  | 7.618 (7.572) | 0.318 |  |
| Sex*anxiety | 2.025 (4.572) | 0.658 |  | -13.885 (11.027) | 0.213 |  |
| **PAM avoidance** |  |  |  |  |  |  |
| *Step 1* |  |  |  |  |  |  |
| Constant | -0.041 (6.805) | 0.995 | 0.047 | -6.053 (14.773) | 0.683 | 0.145 |
| Age | 0.177 (0.126) | 0.161 |  | -0.161 (0.231) | 0.489 |  |
| Illness duration | 0.010 (0.013) | 0.446 |  | 0.022 (0.026) | 0.404 |  |
| Education | 1.487 (1.703) | 0.384 |  | 0.832 (3.692) | 0.822 |  |
| Childhood trauma | 0.267 (0.102) | 0.010** |  | 0.629 (0.218) | 0.005** |  |
| *Step 2* |  |  |  |  |  |  |
| Constant | -0.955 (7.615) | 0.900 | 0.049 | 2.361 (17.772) | 0.895 | 0.159 |
| Age | 0.162 (0.128) | 0.208 |  | -0.169 (0.234) | 0.473 |  |
| Illness duration | 0.010 (0.013) | 0.444 |  | 0.028 (0.027) | 0.307 |  |
| Education | 1.365 (1.725) | 0.430 |  | -0.280 (3.885) | 0.943 |  |
| Childhood trauma | 0.237 (0.114) | 0.039* |  | 0.609 (0.232) | 0.011** |  |
| Sex | 1.033 (2.367) | 0.663 |  | -2.994 (4.682) | 0.525 |  |
| Avoidance | 1.585 (2.420) | 0.513 |  | 3.793 (4.818) | 0.434 |  |
| *Step 3* |  |  |  |  |  |  |
| Constant | -5.341 (8.376) | 0.524 | 0.056 | 6.967 (19.285) | 0.719 | 0.164 |
| Age | 0.153 (0.128) | 0.233 |  | -0.170 (0.235) | 0.473 |  |
| Illness duration | 0.009 (0.013) | 0.505 |  | 0.031 (0.028) | 0.262 |  |
| Education | 1.139 (1.732) | 0.512 |  | 0.114 (3.952) | 0.977 |  |
| Childhood trauma | 0.261 (0.115) | 0.025* |  | 0.598 (0.234) | 0.013* |  |
| Sex | 2.989 (2.835) | 0.293 |  | -5.391 (6.042) | 0.376 |  |
| Avoidance | 13.821 (10.086) | 0.172 |  | -9.060 (20.891) | 0.666 |  |
| Sex*avoidance | -5.615 (4.493) | 0.213 |  | 5.943 (9.396) | 0.529 |  |
|  |  | | | | |  |
| **Personal recovery (RAS)** | | | | | | |
|  | **3-month follow up** | | | **48-month follow up** | | |
| **PAM anxiety** | β (SE) | *p-value* | R^2^ | β (SE) | *p-value* | R^2^ |
| *Step 1* |  |  |  |  |  |  |
| Constant | 4.438 (0.207) | <0.001 | 0.117 | 4.143 (0.551) | <0.001 | 0.117 |
| Age | -0.010 (0.004) | 0.011* |  | 0.001 (0.009) | 0.954 |  |
| Illness duration | 0.000 (0.000) | 0.717 |  | 0.002 (0.005) | 0.697 |  |
| Education | 0.095 (0.053) | 0.073 |  | 0.020 (0.140) | 0.889 |  |
| Childhood trauma | -0.012 (0.003) | <0.001** |  | -0.006 (0.008) | 0.439 |  |
| *Step 2* |  |  |  |  |  |  |
| Constant | 4.755 (0.205) | <0.001 | 0.220 | 4.356 (0.624) | <0.001 | 0.069 |
| Age | -0.010 (0.004) | 0.005** |  | 0.000 (0.009) | 0.958 |  |
| Illness duration | 0.000 (0.000) | 0.984 |  | 0.003 (0.005) | 0.517 |  |
| Education | 0.128 (0.051) | 0.012** |  | -0.035 (0.144) | 0.807 |  |
| Childhood trauma | -0.007 (0.003) | 0.028* |  | -0.010 (0.009) | 0.256 |  |
| Sex | 0.055 (0.069) | 0.427 |  | 0.277 (0.179) | 0.129 |  |
| Anxiety | -0.349 (0.068) | <0.001** |  | -0.038 (0.201) | 0.852 |  |
| *Step 3* |  |  |  |  |  |  |
| Constant | 4.811 (0.222) | <0.001 | 0.222 | 3.832 (0.723) | <0.001 | 0.110 |
| Age | -0.010 (0.004) | 0.006 |  | 0.000 (0.009) | 0.988 |  |
| Illness duration | 0.000 (0.000) | 0.979 |  | 0.005 (0.005) | 0.326 |  |
| Education | 0.130 (0.051) | 0.011** |  | -0.054 (0.143) | 0.706 |  |
| Childhood trauma | (-0.007 (0.003) | 0.026* |  | -0.010 (0.009) | 0.265 |  |
| Sex | (-0.097 (0.238) | 0.683 |  | 1.245 (0.719) | 0.091 |  |
| Anxiety | (-0.384 (0.085) | <0.001** |  | 0.265 (0.295) | 0.374 |  |
| Sex*anxiety | 0.087 (0.130) | 0.504 |  | -0.550 (0.396) | 0.172 |  |
| **PAM avoidance** |  |  |  |  |  |  |
| *Step 1* |  |  |  |  |  |  |
| Constant | 4.427 (0.205) | <0.001** | 0.112 | 4.143 (0.551) | <0.001 | 0.017 |
| Age | -0.009 (0.004) | 0.015 |  | 0.001 (0.009) | 0.954 |  |
| Illness duration | 0.000 (0.000) | 0.287 |  | 0.002 (0.005) | 0.697 |  |
| Education | 0.091 (0.051) | 0.079 |  | 0.020 (0.140) | 0.889 |  |
| Childhood trauma | -0.012 (0.003) | <0.001** |  | -0.006 (0.008) | 0.439 |  |
| *Step 2* |  |  |  |  |  |  |
| Constant | 4.989 (0.212) | <0.001 | 0.244 | 4.395 (0.677) | <0.001 | 0.070 |
| Age | -0.008 (0.004) | 0.034* |  | 0.000 (0.010) | 0.978 |  |
| Illness duration | 0.000 (0.000) | 0.640 |  | 0.004 (0.005) | 0.487 |  |
| Education | 0.076 (0.048) | 0.117 |  | -0.042 (0.147) | 0.778 |  |
| Childhood trauma | -0.004 (0.003) | 0.194 |  | -0.010 (0.009) | 0.248 |  |
| Sex | 0.006 (0.068) | 0.927 |  | 0.275 (0.177) | 0.128 |  |
| Avoidance | -0.397 (0.066) | <0.001** |  | -0.044 (0.182) | 0.811 |  |
| *Step 3* |  |  |  |  |  |  |
| Constant | 5.047 (0.234) | <0.001 | 0.246 | 3.935 (0.770) | <0.001 | 0.102 |
| Age | -0.007 (0.004) | 0.037* |  | 0.001 (0.010) | 0.924 |  |
| Illness duration | 0.000 (0.000) | 0.675 |  | 0.004 (0.005) | 0.426 |  |
| Education | 0.079 (0.048) | 0.106 |  | -0.047 (0.146) | 0.750 |  |
| Childhood trauma | -0.005 (0.003) | 0.168 |  | -0.009 (0.009) | 0.280 |  |
| Sex | -0.155 (0.282) | 0.582 |  | 1.216 (0.785) | 0.129 |  |
| Avoidance | -0.423 (0.080) | <0.001** |  | 0.141 (0.235) | 0.553 |  |
| Sex*avoidance | 0.074 (0.126) | 0.555 |  | -0.424 (0.345) | 0.226 |  |
| Step 1: covariates age, illness duration, education, and childhood trauma entered  Step 2: attachment style additionally entered  Step 3: sex*attachment style interaction effects additionally entered  Abbreviations: PANSS = Positive and Negative Syndrome Scale; WHODAS = WHO Disability Assessment Schedule ; RAS = Recovery Assessment Scale; R^2^=coefficient of determination.  * p-value <0.05 ** p-value <0.0125. considering correction for multiple testing. | | | | | | |

**Supplementary Table S6:** Overview of patients included in the prospective analyses regarding the association between baseline attachment style and change in recovery outcomes over time (between 3 and 48 months follow up).

|  | **Attachment anxiety** | | **Attachment avoidance** | |
| --- | --- | --- | --- | --- |
|  | *Male* | *Female* | *Male* | *Female* |
| Change in symptomatic recovery | 48 | 29 | 50 | 30 |
| Change in social recovery: getting along | 51 | 31 | 53 | 32 |
| Change in social recovery: participation | 51 | 31 | 53 | 32 |
| Change in personal recovery | 31 | 24 | 31 | 25 |
| Data in N.  Recovery outcome measure change between 3 and 48-months follow up. | | | | |

**Supplementary Table S7.** Sex differences in the prospective association between baseline attachment style and change in recovery outcome measures over time whilst correcting for covariates.

| **Change in symptomatic recovery (PANSS)** | | | |
| --- | --- | --- | --- |
| **PAM anxiety (N=77)** | β (SE) | *p-value* | *R^2^* |
| *Step 1* |  |  |  |
| Constant | 7.955 (10.423) | 0.448 | 0.075 |
| Age | -0.012 (0.155) | 0.939 |  |
| Illness duration | 0.167 (0.091) | 0.072 |  |
| Education | -2.295 (2.578) | 0.377 |  |
| Childhood trauma | -0.092 (0.150) | 0.542 |  |
| *Step 2* |  |  |  |
| Constant | 6.218 (12.222) | 0.613 | 0.106 |
| Age | 0.010 (0.157) | 0.951 |  |
| Illness duration | 0.152 (0.094) | 0.110 |  |
| Education | -1.509 (2.661) | 0.573 |  |
| Childhood trauma | -0.019 (0.161) | 0.906 |  |
| Sex | -4.475 (3.325) | 0.184 |  |
| Anxiety | -0.781 (3.590) | 0.828 |  |
| *Step 3* |  |  |  |
| Constant | 13.430 (13.965) | 0.340 | 0.124 |
| Age | 0.001 (0.157) | 0.995 |  |
| Illness duration | 0.140 (0.094) | 0.144 |  |
| Education | -1.154 (2.678) | 0.668 |  |
| Childhood trauma | -0.059 (0.165) | 0.724 |  |
| Sex | -17.764 (12.929) | 0.175 |  |
| Anxiety | -4.416 (4.954) | 0.377 |  |
| Sex*anxiety | 7.626 (7.171) | 0.292 |  |
| **PAM avoidance (N=80)** |  |  |  |
| *Step 1* |  |  |  |
| Constant | 12.484 (10.327) | 0.231 | 0.023 |
| Age | -0.032 (0.158) | 0.839 |  |
| Illness duration | -0.007 (0.018) | 0.707 |  |
| Education | -2.558 (2.616) | 0.332 |  |
| Childhood trauma | -0.103 (0.153) | 0.501 |  |
| *Step 2* |  |  |  |
| Constant | 4.952 (12.371) | 0.690 | 0.060 |
| Age | -0.015 (0.159) | 0.924 |  |
| Illness duration | -0.012 (0.018) | 0.497 |  |
| Education | -1.453 (2.711) | 0.594 |  |
| Childhood trauma | -0.053 (0.161) | 0.745 |  |
| Sex | -4.557 (3.352) | 0.179 |  |
| Avoidance | 2.076 (3.149) | 0.512 |  |
| *Step 3* |  |  |  |
| Constant | 14.332 (13.177) | 0.281 | 0.112 |
| Age | -0.018 (0.156) | 0.907 |  |
| Illness duration | -0.005 (0.018) | 0.774 |  |
| Education | -0.719 (2.689) | 0.790 |  |
| Childhood trauma | -0.083 (0.159) | 0.602 |  |
| Sex | -28.969 (13.791) | 0.040* |  |
| Avoidance | -2.597 (4.014) | 0.520 |  |
| Sex*avoidance | 11.331 (6.217) | 0.074 |  |
|  | | |  |
| **Change in social functioning: getting along (WHODAS 2.0)** | | | |
| **PAM anxiety (N=82)** | β (SE) | *p-value* | *R^2^* |
| *Step 1* |  |  |  |
| Constant | 6.641 (17.453) | 0.705 | 0.113 |
| Age | -0.361 (0.265) | 0.178 |  |
| Illness duration | 0.288 (0.155) | 0.068 |  |
| Education | -3.296 (4.264) | 0.442 |  |
| Childhood trauma | 0.264 (0.250) | 0.295 |  |
| *Step 2* |  |  |  |
| Constant | 6.418 (20.661) | 0.757 | 0.123 |
| Age | -0.346 (0.270) | 0.205 |  |
| Illness duration | 0.266 (0.162) | 0.105 |  |
| Education | -2.485 (4.451) | 0.579 |  |
| Childhood trauma | 0.332 (0.269) | 0.221 |  |
| Sex | -4.339 (5.580) | 0.440 |  |
| Anxiety | -1.432 (6.226) | 0.819 |  |
| *Step 3* |  |  |  |
| Constant | -0.794 (23.732) | 0.973 | 0.128 |
| Age | -0.334 (0.272) | 0.224 |  |
| Illness duration | 0.280 (0.164) | 0.094 |  |
| Education | -2.905 (4.523) | 0.523 |  |
| Childhood trauma | 0.369 (0.276) | 0.187 |  |
| Sex | 9.430 (22.646) | 0.679 |  |
| Anxiety | 2.278 (8.608) | 0.792 |  |
| Sex*anxiety | -7.868 (12.536) | 0.533 |  |
| **PAM avoidance (N=85)** |  |  |  |
| *Step 1* |  |  |  |
| Constant | 17.398 (17.978) | 0.337 |  |
| Age | -0.415 (0.282) | 0.146 |  |
| Illness duration | -0.096 (0.032) | 0.004** |  |
| Education | 4.188 (4.505) | 0.356 |  |
| Childhood trauma | 0.243 (0.267) | 0.365 |  |
| *Step 2* |  |  |  |
| Constant | 12.774 (21.768) | 0.559 |  |
| Age | -0.399 (0.287) | 0.169 |  |
| Illness duration | -0.100 (0.033) | 0.004** |  |
| Education | -3.193 (4.758) | 0.505 |  |
| Childhood trauma | 0.288 (0.284) | 0.314 |  |
| Sex | -4.307 (5.901) | 0.468 |  |
| Avoidance | 0.908 (5.734) | 0.875 |  |
| *Step 3* |  |  |  |
| Constant | 22.462 (23.472) | 0.342 |  |
| Age | -0.402 (0.286) | 0.166 |  |
| Illness duration | -0.093 (0.034) | 0.008** |  |
| Education | -2.365 (4.810) | 0.625 |  |
| Childhood trauma | 0.267 (0.284) | 0.352 |  |
| Avoidance | -31.341 (25.426) | 0.222 |  |
| Sex | -4.136 (7.353) | 0.576 |  |
| Sex*avoidance | 12.499 (11.436) | 0.279 |  |
|  | | |  |
| **Change in social functioning: participation (WHODAS 2.0)** | | | |
| **PAM anxiety (N=82)** | β (SE) | *p-value* | *R^2^* |
| *Step 1* |  |  |  |
| Constant | -12.475 (16.103) | 0.441 | 0.046 |
| Age | -0.132 (0.244) | 0.592 |  |
| Illness duration | 0.158 (0.143) | 0.274 |  |
| Education | -0.117 (3.934) | 0.976 |  |
| Childhood trauma | 0.268 (0.230) | 0.249 |  |
| *Step 2* |  |  |  |
| Constant | -5.723 (19.070) | 0.765 | 0.056 |
| Age | -0.143 (0.249) | 0.568 |  |
| Illness duration | 0.129 (0.149) | 0.393 |  |
| Education | -0.094 (4.109) | 0.982 |  |
| Childhood trauma | 0.324 (0.248) | 0.196 |  |
| Sex | -0.702 (5.151) | 0.892 |  |
| Anxiety | -4.453 (5.747) | 0.441 |  |
| *Step 3* |  |  |  |
| Constant | -11.483 (21.923) | 0.602 | 0.061 |
| Age | -0.133 (0.251) | 0.598 |  |
| Illness duration | 0.140 (0.152) | 0.361 |  |
| Education | -0.430 (4.179) | 0.918 |  |
| Childhood trauma | 0.353 (0.255) | 0.171 |  |
| Anxiety | 10.294 (20.920) | 0.624 |  |
| Sex | -1.490 (7.952) | 0.852 |  |
| Sex*anxiety | -6.283 (11.581) | 0.589 |  |
| **PAM avoidance (N=85)** |  |  |  |
| *Step 1* |  |  |  |
| Constant | -8.797 (15.594) | 0.575 | 0.043 |
| Age | -0.158 (0.245) | 0.520 |  |
| Illness duration | 0.021 (0.028) | 0.448 |  |
| Education | -0.334 (3.908) | 0.932 |  |
| Childhood trauma | 0.261 (0.231) | 0.264 |  |
| *Step 2* |  |  |  |
| Constant | -6.140 (18.946) | 0.747 | 0.045 |
| Age | -0.150 (0.250) | 0.549 |  |
| Illness duration | 0.022 (0.029) | 0.445 |  |
| Education | -0.343 (4.141) | 0.934 |  |
| Childhood trauma | 0.284 (0.247) | 0.254 |  |
| Seks | -0.775 (5.137) | 0.880 |  |
| Avoidance | -1.600 (4.991) | 0.750 |  |
| *Step 3* |  |  |  |
| Constant | 1.535 (20.463) | 0.940 | 0.060 |
| Age | -0.152 (0.250) | 0.544 |  |
| Illness duration | 0.028 (0.030) | 0.344 |  |
| Education | 0.312 (4.194) | 0.941 |  |
| Childhood trauma | 0.267 (0.248) | 0.285 |  |
| Avoidance | -5.596 (6.411) | 0.386 |  |
| Sex | -22.194 (22.167) | 0.321 |  |
| Sex*avoidance | 9.903 (9.970) | 0.324 |  |
|  | | |  |
| **Change in personal recovery (RAS)** | | | |
| **PAM anxiety (N=55)** | β (SE) | *p-value* | *R^2^* |
| *Step 1* |  |  |  |
| Constant | 0.116 (0.662) | 0.862 | 0.043 |
| Age | 0.004 (0.011) | 0.697 |  |
| Illness duration | 0.005 (0.006) | 0.441 |  |
| Education | -0.137 (0.170) | 0.424 |  |
| Childhood trauma | 0.004 (0.010) | 0.687 |  |
| *Step 2* |  |  |  |
| Constant | -0.108 (0.733) | 0.883 | 0.134 |
| Age | 0.001 (0.011) | 0.895 |  |
| Illness duration | 0.008 (0.006) | 0.178 |  |
| Education | -0.197 (0.171) | 0.257 |  |
| Childhood trauma | -0.004 (0.010) | 0.699 |  |
| Sex | 0.308 (0.211) | 0.152 |  |
| Anxiety | 0.311 (0.236) | 0.195 |  |
| *Step 3* |  |  |  |
| Constant | -0.853 (0.839) | 0.315 | 0.192 |
| Age | 0.002 (0.011) | 0.822 |  |
| Illness duration | 0.011 (0.006) | 0.082 |  |
| Education | -0.228 (0.169) | 0.184 |  |
| Childhood trauma | -0.004 (0.010) | 0.726 |  |
| Anxiety | 1.697 (0.838) | 0.049* |  |
| Sex | 0.744 (0.343) | 0.036* |  |
| Sex*anxiety | -0.787 (0.460) | 0.095 |  |
| **PAM avoidance (N=56)** |  |  |  |
| *Step 1* |  |  |  |
| Constant | 0.116 (0.662) | 0.862 | 0.043 |
| Age | 0.004 (0.011) | 0.697 |  |
| Illness duration | 0.005 (0.006) | 0.441 |  |
| Education | -0.137 (0.170) | 0.424 |  |
| Childhood trauma | 0.004 (0.010) | 0.687 |  |
| *Step 2* |  |  |  |
| Constant | -0.210 (0.814) | 0.798 | 0.124 |
| Age | 0.001 (0.011) | 0.964 |  |
| Illness duration | 0.007 (0.006) | 0.249 |  |
| Education | -0.154 (0.179) | 0.396 |  |
| Childhood trauma | -0.003 (0.010) | 0.779 |  |
| Sex | 0.323 (0.212) | 0.135 |  |
| Avoidance | 0.248 (0.222) | 0.271 |  |
| *Step 3* |  |  |  |
| Constant | -0.617 (0.943) | 0.516 | 0.140 |
| Age | 0.001 (0.011) | 0.904 |  |
| Illness duration | 0.008 (0.006) | 0.222 |  |
| Education | -0.153 (0.180) | 0.400 |  |
| Childhood trauma | -0.002 (0.010) | 0.819 |  |
| Avoidance | 1.114 (0.940) | 0.243 |  |
| Sex | 0.411 (0.292) | 0.167 |  |
| Sex*avoidance | -0.358 (0.414) | 0.393 |  |
| Attachment anxiety and avoidance at follow up = 6 months after baseline.  Change in recovery outcome measures between 48 months follow up and 3 months follow up.  Step 1: covariates age, illness duration, education, and childhood trauma entered; step 2: attachment style; step 3: sex*attachment style interaction effects entered.  * p-value <0.05 ** p-value <0.0125, considering correction for multiple testing. | | | |
